# Organic Iron Compounds

**Published:** 1909-08-14

**Authors:** 


### THERAPEUTICS.

### ORGANIC IRON COMPOUNDS.

*Ferratin.*—This is an artificially-prepared substance containing iron in combination with an organic acid, and is said to resemble in composition a natural compound of iron and protein isolated by Schmiedeberg and Marfori from the pig's liver. Its iron-content amounts to 6 per cent. The solution of ferratin called ferratose is a dark brown fluid, the taste of which is not unpleasant. Its composition is ferratin, glycerine, alcohol, and angostura, made up with distilled water; one tablespoonful contains .75 grain iron. The ordinary three doses daily therefore correspond to about 2 grains. The indication for the use of this compound is anæmia, either primary or secondary. It has been somewhat extensively used on the Continent, where the advantage of giving iron in the form of an organic compound seems very generally recognised.

*Triferrin* is known as paranucleinate of iron, and is prepared by precipitating the organic phosphorus compound obtained from a pepsin digest of casein. It is thus iron combined with a form of phosphoprotein or pseudonuclein. It contains—

| | |
|---|---|
| Nitrogen ... ... ... ... | 9 per cent. |
| Phosphorus ... ... ... | 2.5 per cent. |
| $Fe_2O_3$ ... ... ... ... ... | 22 per cent. |

It is insoluble in weak hydrochloric acid, and is thus unacted on by gastric digestion; in the small intestine, however, it is readily dissolved by the alkaline medium. Clinical observations show that triferrin is very well tolerated by persons with weak digestion. The usual dose is 4½ grains thrice daily. Tablets containing this amount and flavoured with chocolate are on the market, and are prepared by Messrs. Knoll and Co.

*Ferropyrin* is a compound of perchloride of iron with antipyrin, and is an orange-red powder, forming a red solution in five parts of water. It may be employed in place of the ordinary tinct. ferri perchloridi, both externally and internally. The advantages of this preparation are that it is more powerfully hæmostatic, is not caustic, and does not clog the wound or dressings. It may also be found of value in hæmorrhage from the gums after extractions and in gastric and intestinal bleeding. For gastric conditions the following formula may be employed:—

| | | |
|---|---|---|
| ℞ | Ferropyrini ... ... ... ... | gr. 15 |
| | Syr. aurant ... ... ... ... | ʒij. |
| | Aq. ad ... ... ... ... ... ... | ℥iv. |
| | *Ft. mist.* | |

*Sig.*: One half at once, the remainder in two hours.

In chlorosis it is said to be particularly useful in cases complicated by dyspepsia and neuralgia, where the anodyne properties of the antipyrin are needed. It is well absorbed, and may be given thus:—

| | | |
|---|---|---|
| ℞ | Ferropyrini ... ... ... ... | gr. 10 |
| | Acidi hydrochlor. dil. ... ... ... | ♏v. |
| | Glycerini pepsini ... ... ... ... | ℥j. |
| | Aq. ad ... ... ... ... ... ... | ℥vij. |
| | *Ft. mist.* | |

*Sig.*: ℥ss. thrice daily after food.

Ferropyrin is prepared by Knoll and Co.

*Iron somatose* consists of the albumoses derived from beef in somewhat firm chemical combination with iron. The latter is intended to influence the blood, whilst the somatose has nutritive properties and is a stimulant to gastric digestion. Iron somatose is a light brown, nearly tasteless, and odourless powder, dissolving readily in water. It contains 2 per cent. of iron, which is not separated by the action of dilute acids in the stomach. It has no disturbing action on digestion, can be taken for long periods, and is well tolerated by children. It does not blacken the teeth, and is not constipating. Improvement of appetite and increase in weight are observed to occur rapidly. It is indicated in all conditions for which iron is usually given. As to the method of administration, iron somatose is best given in small divided doses, as these are more thoroughly assimilated. The powder must invariably be dissolved, and the solution may be added to milk, hot soup or beef-tea, cocoa, white wine or beer. It should not be mixed with tea, coffee, or red wine, as the contained tannin precipitates the albumoses. The solution should be prepared by making the powder first into a thin paste, and then stirring in hot water, or the powder may be sprinkled on the surface of the fluid and left to stand until complete solution has taken place. One to two teaspoonfuls per diem in divided doses represent .2 to .3 gram of iron, or nine to ten Blaud's pills. Iron somatose is prepared by Messrs. Bayer and Co.

